# Detection of Prostate Cancer Metastases During Pelvic Lymph Node Dissection with the PSMA-Targeted Fluorescent Agent OTL78: A Phase II Study

**DOI:** 10.1007/s11307-025-02052-x

**Published:** 2025-10-09

**Authors:** Judith A. Stibbe, Daan F. G. Linders, Robin A. Faber, Rob F. M. Bevers, Erik J. van Gennep, Stijn A. S. L. P. Crobach, Shadhvi S. Bhairosingh, Timothy G. Biro, Prof Jacobus Burggraaf, Prof Alexander L. Vahrmeijer

**Affiliations:** 1 Department of Surgery, Leiden University Medical Center, Leiden, 2333 ZA The Netherlands; 2 Center for Human Drug Research, Leiden, 2333 CL The Netherlands; 3 Department of Surgery, Noordwest Ziekenhuisgroep, Alkmaar, 1815 JD The Netherlands; 4 Department of Otorhinolaryngology, Leiden University Medical Center, Leiden, 2333 ZA The Netherlands; 5 Department of Urology, Leiden University Medical Center, Leiden, 2333 ZA The Netherlands; 6 Department of Pathology, Leiden University Medical Center, Leiden, 2333 ZA The Netherlands; 7 On Target Laboratories, West Lafayette, IN USA

**Keywords:** Fluorescence, Lymph node metastases, OTL78, Pelvic lymph node dissection, Prostate cancer, PSMA

## Abstract

**Purpose:**

This study aimed to assess the feasibility of intraoperative fluorescence imaging using the PSMA-targeted fluorescent tracer OTL78 for detecting lymph node metastases during pelvic lymph node dissection (PLND) in patients undergoing staging or salvage surgery for prostate cancer.

**Procedures:**

In a prospective pilot study, six patients scheduled for robot-assisted PLND received a single intravenous infusion of OTL78 at a dose of either 0.06 mg/kg or 0.03 mg/kg, administered 1–2 h prior to surgery. Intraoperatively, lymph node clusters were evaluated using fluorescence imaging. Post-surgical histopathological analysis and immunohistochemistry were performed to confirm tumor presence and PSMA overexpression in fluorescent nodes. The primary outcome was the feasibility of fluorescence imaging in detecting metastatic lymph nodes during PLND.

**Results:**

Fluorescence imaging demonstrated a sensitivity of 66.7% and specificity of 91.7% for identifying metastatic lymph nodes. The positive predictive value was 66.7%, and the negative predictive value was 91.7%. Metastasized lymph nodes (MLN) exhibited significantly higher median fluorescence intensity (MFI) than benign lymph nodes (BLN): 0.51 [IQR 0.11–0.74] vs. 0.06 [IQR 0.03–0.12], p = 0.024. Immunohistochemistry confirmed PSMA overexpression in fluorescent malignant regions. No adverse reactions to the tracer were reported.

**Conclusions:**

Intraoperative fluorescence imaging with the tracer OTL78 is a feasible technique for identifying metastatic lymph nodes during PLND. Fluorescence guidance may assist in detecting small metastatic deposits within nodal clusters that are otherwise difficult to localize. Larger studies are needed to validate these findings and optimize the imaging protocol for broader clinical use.

## Introduction

Pelvic lymph nodes (LNs) represent the most common site for regional metastases in men diagnosed with prostate cancer (PCa)[1]. Lymph node invasion (LNI) has been shown to be an adverse prognostic factor for biochemical recurrence and survival[2, 3]. Due to the limited sensitivity of currently available imaging techniques, pelvic lymph node dissection (PLND) still is the best staging method for detecting LNI[4]. To date, a therapeutic effect of PLND has not been demonstrated, yet the substantial difference in PCa-related death between patients with extensive and limited LNI suggests PLND might improve oncological outcomes in certain subgroups[5, 6]. The intraoperative identification and removal of all pelvic LN during PLND is challenging and hardly feasible because of the time and extent of surgery, risk of complications, and the associated costs. For practical reasons, only the areas with the highest chance are sampled, potentially resulting in understaging and suboptimal oncological outcomes[7]. Therefore, enhanced detection tools for intraoperative visualization of metastasized LN during PLND are warranted. Tumor-targeted near-infrared (NIR) fluorescence imaging is a technique that combines the administration of a fluorescence contrast agent directed towards tumor-specific molecular targets with the use of a camera system that can detect NIR fluorescence light (700–900 nm)[8]. It allows for real-time intraoperative optical imaging by selectively highlighting cells that overexpress certain molecular targets. In PCa, the prostate specific membrane antigen (PSMA) is an excellent target for fluorescence-guided surgery. PSMA is a transmembrane glycoprotein, predominantly expressed on the cell membrane of prostate cells, that seems to play a role in prostate growth and differentiation[9]. Binding of a substrate to PSMA leads to rapid internalization through clathrin-mediated endocytosis[10]. PSMA expression has been shown to increase with advancing cellular dysplasia, resulting in a 100- to 1000-fold overexpression on PCa cells that is preserved in metastases[11–14]. A promising NIR imaging agent in PCa is OTL78, a small molecule directed against PSMA labeled with a fluorescent dye. It consists of the high-affinity PSMA-targeting ligand 2-[3-(1,3-dicarboxypropyl)-ureido]pentanedioic acid (DUPA), a 14 atoms long polyethylene glycol-dipeptide linker, and an NIR cyanine dye[15, 16]. OTL78 has a maximum excitation and emission wavelengths of 776 nm and 793 nm respectively. (Pre)clinical studies demonstrated that OTL78 allows for tumor visualization with satisfactory tumor-to-background ratio (TBR), indicating that it could be a suitable agent for intraoperative detection of pelvic LN metastases of PCa[17, 18]. The aim of the proof of concept phase II clinical trial (NL8552) was to assess the feasibility of OTL78 for imaging of pelvic LN metastases in PCa patients undergoing PLND.

## Patients and Methods

### Patients

Six male patients aged 18 years or older were enrolled in the study, all fit for surgery and able and willing to comply with study procedures, with a 12-lead ECG and clinical laboratory test results within normal limits. All patients were scheduled to undergo PLND for salvage or staging because of known primary PCa (confirmed by histopathology) or high suspicion of recurrent PCa with at least an N1 status on PSMA-PET scan at the Leiden University Medical Center (LUMC). Patients with laboratory results implicating impaired renal or liver function; significant allergies, or previous participation in an OTL study were excluded.

### OTL78 Administration and Surgery

Prior to surgery, patients received a single dose of OTL78 (On Target Laboratories, West Lafayette, Indiana, USA) intravenously. The first three patients received a dose of 0.06 mg/kg, next three received a dose of 0.03 mg/kg. The dose-imaging window was 2 h. The selected doses and the dose-imaging window were based on animal toxicology as well as fluorescence studies in mice and a safety study in healthy volunteers. The relatively short dose-imaging interval was the briefest that could be implemented in the clinical workflow. Fluorescence results prompted dose reduction. Patients were monitored for at least 24 h postoperatively according to standard clinical practice. Adverse events were collected up to 2 weeks after discharge. Surgery was performed robotically-assisted (Da Vinci Xi, Intuitive, CA, USA) by two experienced urologists per standard of care. The NIR fluorescence laparoscopic camera system Quest Spectrum Platform 2 (Quest Medical Imaging, Middenmeer, Noord-Holland, The Netherlands) was used for imaging.

### Fluorescence Imaging

During PLND, all lymph node clusters (LNCs) were imaged *in vivo* before resection. This did not significantly disrupt the workflow, as the laparoscopic ports used for surgery could also be used for imaging. Only the Da Vinci arm had to be temporarily repositioned to allow placement of the Quest system's laparoscopic camera. Imaging was performed using short videos or snapshots via the 800 nm channel of the Quest system, taking only a few seconds. Images were saved and stored on a secure drive for post hoc analysis. LNCs were put in separate containers and sent to the pathologist who separated LN from LNCs, followed by lamination, cassette insertion and formalin fixation. The laminated LNs were imaged with the NIR fluorescence imaging system Pearl Trilogy (LI-COR, Lincoln, Nebraska, USA). Cassettes were embedded in paraffin for histological assessment.

### Microscopic Assessments

At least three sequential 4 μm slides were requested from all formalin-fixed-paraffin-embedded (FFPE) LNs originating from a tumor positive and/or fluorescent LNC. Per LN, one slide was stained with hematoxylin and eosin (HE) and digitized using the Panoramic 250 Flash III scanner (3D Histech, Budapest, Hungary). These images were examined by a board-certified uropathologist, who delineated tumor-positive regions while blinded for fluorescence. Another slide was deparaffinized using xylene and rehydrated in decreasing concentrations of ethanol. Subsequently, slides were rinsed with demineralized water and endogenous peroxidase was blocked with 0.3% hydrogen peroxide (Merck Millipore, Darmstadt, Germany) for 20 min at room temperature. Slides were rinsed with demineralized water and antigen retrieval was performed in the PT Link (Agilent, Santa Clara, USA), using Target Retrieval Solution pH 6.0 (Agilent, Santa Clara, USA) at 95 °C. After rinsing with phosphate buffered saline (PBS, pH 7.4), slides were stained overnight at room temperature with the primary antibodies, diluted in 1% bovine serum albumin/PBS, against PSMA (clone 3E6 DAKO, dilution 1:200). After three PBS washing steps, the slides were incubated with HRP-labelled secondary antibodies against mouse antibodies (EnVision, Agilent, Santa Clara, USA) for 30 min at room temperature. Binding of the antibodies was visualized using 3,3’-diaminobenzidine (Agilent, Santa Clara, USA). All slides were counterstained with hematoxylin for 10–15 s, dehydrated at 37 °C and mounted with pertex. The immunohistochemical staining was digitized by scanning of the slides using the Pannoramic 250 Flash III scanner (3D Histech, Budapest, Hungary). One plain tissue slide per requested FFPE LN was scanned using the NIR fluorescence sensitive Odyssey CLx (LI-COR, Lincoln, Nebraska, USA).

### Fluorescence Signal Analysis

The intraoperative fluorescence images of each identified and resected LNC was analyzed using ImageJ v1.53 h software (National Institutes of Health, Bethesda, Maryland, USA). One region of interest (ROI) was drawn around the strongest fluorescence signal in the LNC (*signal)* and one around surrounding (adipose) tissue (*background*). The mean fluorescence intensity (MFI), defined as the fluorescent signal divided by the number of pixels, of both ROIs constituted the tumor-to-background ratio (TBR) or in case of benign LNCs, the signal-to-background ratio (SBR). LNCs that showed an *in vivo* TBR ≥ 1.5 were defined as fluorescence-positive and fluorescence-negative with TBR < 1.5. The ImageStudio software (LI-COR, Lincoln, Nebraska, USA) was used for MFI calculations of the whole LN in cassettes and the (PA confirmed) malignant areas in the microscopic slides scanned with the Odyssey.

### Statistical Analysis

Statistical analyses were performed using SPSS version 25.0 software (SPSS, IBM Corporation, NY, USA) and GraphPad Prism 8 (GraphPad Software Inc., La Jolla, CA, USA). Summary measures are given as mean (SD) or median (IQR). To investigate differences in MFI between LNCs and background, the Wilcoxon signed-rank test was used. The Mann–Whitney U test was used to investigate differences in TBR/SBR and MFI between metastasized and benign LNCs and between the two different dosing cohorts and to examine the MFI of individual benign and malignant LNs. All statistical tests were performed two-sided and results were considered statistically significant at the level of *p* < 0.05. Data are summarized in graphs and bar charts generated by GraphPad Prism 9.

## Results

Patient demographics and clinical data are summarized in Table 1. In total, 6 patients with primary or recurrent PCa scheduled to undergo PLND for staging or salvage were enrolled. Median [IQR] age at diagnosis was 71 years [62.3–74.5]. Median [IQR] PSA at PLND was 14.9 ng/ml [1.72–36.80]. All patients had received a PSMA-PET scan and three patients were N0 and underwent PLND for staging purposes. The other three patients were N1 and underwent PLND as salvage surgery.

**Table 1:** Patient demographics and clinical data

Dosing cohort	Patient#	Age at PLND (years)	Indication PLND	N-stage at PSMA-PETCT	Tumor stage at diagnosis	PSA at PLND (ng/ml)	Histopathological N-stage
1	1	57	Salvage	N1^a^	pT3a	0.29	N1
1	2	70	Staging	N0	mT3b	35.4	N0
1	3	74	Salvage	N1^b^	pT2a	2.2	N1
2	4	72	Staging	N0	mT3b	41	N0
2	5	76	Salvage	N1^c^	cT2b	3.3	N0^d^
2	6	64	Staging	N0	mT3a	26.5	N0

Notes: Patient demographics and clinical data are shown. Patients were subdivided in two dosing cohorts; DC1 (0.06 mg/kg) and DC2 (0.03 mg/kg)

*PLND *pelvic lymph node dissection, *PSMA *prostate specific membrane antigen, *PET *positron emission tomography, *PSA *prostate-specific antigen, *LNM *lymph node metastases

^a^ 1 lymph node in total, left iliac region

^b^ 3 lymph nodes in total, 1 in the right iliac region, 2 in the left iliac region

^c^ 1 lymph node in total, right iliac region

^d^ The patient was classified as radiological N1 (cN1); however, the lymph node could not be visualized or resected due to surgical challenges posed by fibrosis. Consequently, the pathological N status was (p)N0

### Fluorescence and Immunohistochemistry

**Fig. 1:**
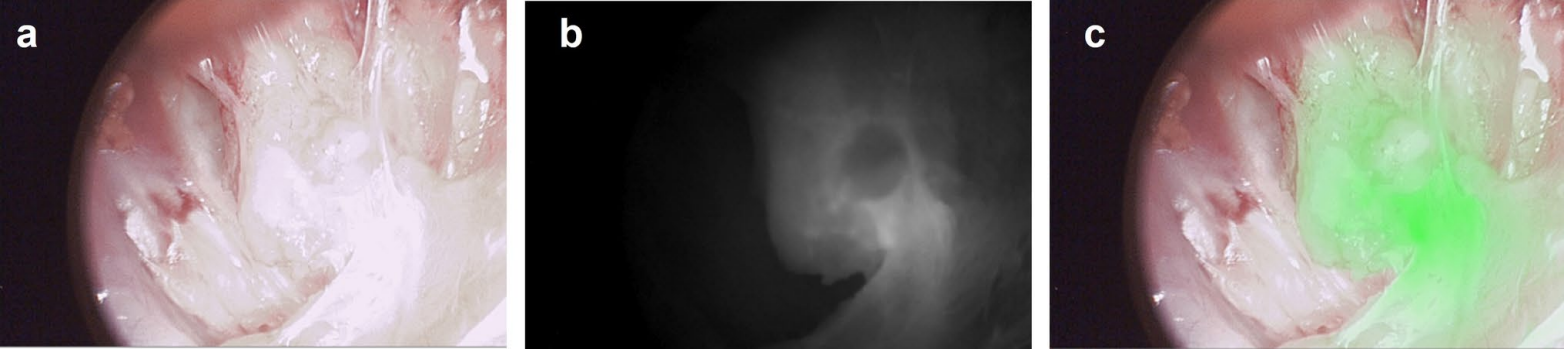
(**a**) White light image of lymph node within a lymph node cluster; (**b**) true fluorescence image (**c**) fluorescence/white light overlay

**Fig. 2:**
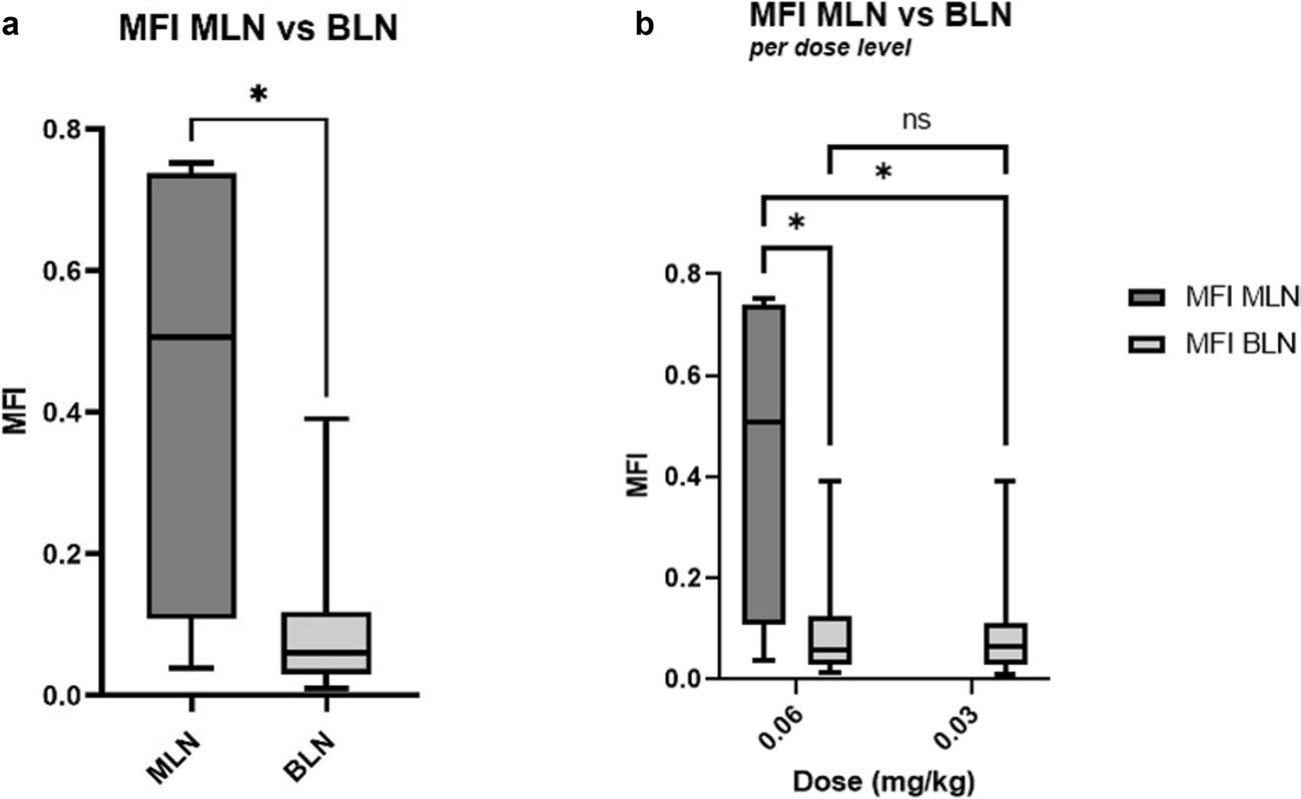
(**a**) *Ex vivo* mean fluorescence intensity of malignant lymph nodes (MLN) versus benign lymph nodes (BLN). (**b**) *Ex vivo* MFI of MLN vs BLN, divided per DC. Box-and-whisker plot shows the minimum, first quartile, second quartile, third quartile, and maximum MFI. *p<0.050

**Fig. 3:**
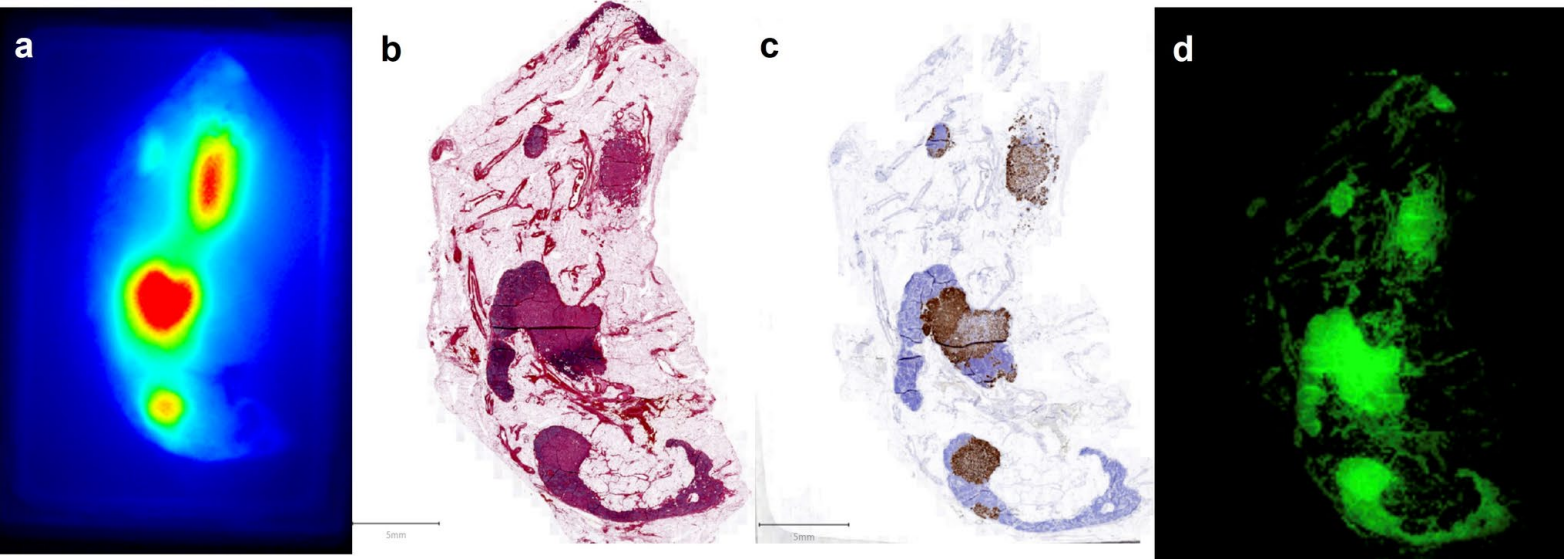
A representative example of a lymph node containing metastases of prostate cancer (**a**) scanned for fluorescence after bisection and formalin fixation; (**b**) scanned after HE staining; (**c**) scanned after PSMA staining; (**d**) scanned for microscopic fluorescence

In total, 16 LNCs were resected (Table 2). Of these, three LNCs contained one or more tumor-positive (metastasized) lymph nodes (MLNs) and two were tumor-positive as well as fluorescent *in vivo* (TBR ≥ 1.5, Fig. 1). One LNC was fluorescent *in vivo* but did not contain tumor-positive LNs (false-positive). In these 6 patients, OTL78 had a sensitivity and positive predictive value of 66.7% and a specificity and negative predictive value of 91.7% for the detection of metastasized LNC *in vivo*. After separation from their clusters, *ex vivo* imaging of LNs in cassettes showed a median MFI [IQR] in benign lymph nodes (BLN) (regardless of dose cohort) of 0.06 [0.03 −0.12], and 0.51 [0.11–0.73] in MLNs (p = 0.024*; Fig. 2A). Median MFI [IQR] of MLN and BLN in DC1 (0.06 mg/kg) was 0.51 [0.11–0.74] and 0.06 [0.03–0.13] respectively (p = 0.028*, Fig. 2B). No MLNs were found in DC2, the median MFI of the BLNs was 0.07 [0.03–0.11], which did not differ from median MFI of BLN in DC1 (p = 0.814, Fig. 2B). Median MFI of MLNs (all DC1) was significantly higher compared to BLNs in DC2 (p = 0.034*, Fig. 2B). The malignant regions within the MLNs could were delineated using fluorescence microscopy (Fig. 3). At fluorescence microscopy, median[IQR] MFI of malignant areas was 38.50 [29.23–454.88] as opposed to 12.75 [7.77–47.75] of benign areas in the MLNs. Median[IQR] TBR on microscopical assessment was 3.62 [2.98–8.58]. Immunohistochemistry confirmed PSMA-overexpression of HE-confirmed and fluorescent malignant regions in the MLNs.

**Table 2:** Fluorescence in lymph node clusters

Patient#	LNC	+ on PSMA-PET/CT	Tumor present	Fluorescent *in vivo*	TBR/SBR *in vivo*	TBR *ex vivo*^a^
1	I	No	No	Yes	1.72	NA
1	II	No	No	No	1.45	NA
1	III	No	No	No	1.47	NA
1	IV	No	No	No	1.41	NA
1	V	Yes	**Yes**	Yes	1.56	10.09
1	VI	No	No	No	2.74^b^	NA
2	I	No	No	NA	NA	NA
2	II	No	No	No	1.05	NA
2	IV	No	No	No	1.23	NA
3	I	Yes	**Yes**	No	1.00	4.07
3	II	Yes	**Yes**	Yes	1.78	2.92
4	I	No	No	No	1.21	NA
4	II	No	No	No	1.47	NA
5	I	Yes	No^c^	No	1.15	NA
6	I	No	No	No	1.46	NA
6	II	No	No	No	1.24	NA
Total	16	4	3	3		

Notes: Shown are the *in vivo* and *ex vivo* intraoperative fluorescence imaging results for each of the 16 resected LNCs. Three of the 16 LNCs contained tumor-positive (metastasized) lymph nodes. Of one LNC (2.I), no *in vivo* fluorescence images were available

*LN *lymph node, *LNC *lymph node cluster, *PSMA *prostate-specific membrane antigen, *SBR *signal-to-background ratio, *TBR *tumor-to-background ratio

^a^ TBR of the tumor region in the malignant and fluorescent node of the LNC (scanned with the Odyssey CLx (LI-COR, Lincoln, Nebraska, USA)

^b^ High SBR due to very low background signal, absolute lymph node fluorescence (MFI) was low

^c^ Positive lymph node on PSMA PET/CT could not be reached intraoperatively due to (radiotherapy-induced) fibrosis

## Discussion

The aim of this proof-of-concept study was to assess the feasibility of OTL78 for imaging of pelvic LN metastases in PCa patients undergoing PLND as staging or salvage surgery. Fluorescence imaging with OTL78 had a sensitivity of 66.7% and a specificity of 91.7% for the detection of MLNs not yet separated from their clusters *in vivo,* which is favorable in comparison to other PSMA-targeted agents such as IR800-IAB2M (64% and 64% respectively)[19]. In contrast to radioguided PSMA-targeted approaches such as ^99mTc-PSMA and hybrid tracers, which offer deep-tissue localization through gamma detection, fluorescence imaging provides high spatial resolution and real-time visual guidance during surgery[20]. These modalities may be viewed as complementary, with fluorescence particularly enhancing intraoperative precision and enabling identification of lesions that might be missed due to tissue coverage or sampling limitations. Implementation of intraoperative fluorescence imaging in patients undergoing PLND for staging or salvaging was feasible and all malignant areas had PSMA-overexpression and were fluorescent *ex vivo*. Intraoperative and postoperative fluorescent signal facilitated the identification of a suspicious LN in a patient (subject 1) who was known to have a PSMA-PET-avid LN. In this instance, *ex vivo* fluorescence proved to be particularly useful, because histopathological examination initially failed to show any malignancy in the fluorescent node. Because of the bright signal, re-evaluation was requested, involving deeper cuts of the fluorescent LN. Subsequently, the more central sections unveiled the previously undetected (e.g. covered with benign tissue) metastasis. Without the fluorescent signal, the patient might have been erroneously assigned a pN0 status, potentially impacting the subsequent course of follow-up care. In subject 3, the preoperative PSMA-PET showed the presence of three avid LNs, suggestive of malignancy. Among these, the node situated on the patient's right side exhibited the highest intensity and largest dimensions on the PSMA-PET scan, with intraoperatively a conspicuously fluorescent appearance. Pathological analysis unveiled a malignant region measuring 14 mm within the fluorescent node, which exhibited persistent fluorescence *ex vivo* and PSMA overexpression. Conversely, the smaller nodes on the left side were not visualized intraoperatively via fluorescence imaging, and initial pathologic examination failed to identify malignancy within the left-sided lymph node clusters. Upon request, residual adipose-like material underwent *ex vivo* fluorescence re-scanning, revealing fluorescent tissue, which was confirmed to harbor two small malignant LNs, with the largest diameter measuring 6 mm. The presence of overlying adipose tissue likely hindered the *in vivo* detection of these diminutive fluorescent metastasized nodes, possibly related to the penetration depth of near-infrared light. This limitation was mitigated by post hoc fluorescence microscopy, which demonstrated that OTL78 accurately delineated all regions exhibiting PSMA overexpression, providing more evidence of OTL78 accumulation in PSMA-expressing tumor cells within LNs. The sample size of this exploratory study was too small to draw final conclusions about the efficacy of *in vivo* imaging of MLNs. For future studies with larger cohorts of patients, it should be considered to only enroll patients planned for salvage lymph node dissection with PSMA-PET avid nodes on preoperative scans. A limitation related to the small sample size, was the absence of patients with MLNs in dose cohort 2. Consequently, fluorescence analyses within this lower dose cohort and comparison with the higher dose could not be done. However, fluorescence imaging with OTL78 *in vivo* and *ex vivo* prompted pathological re-examination and adjusting the conclusion of the pathological report in 2 out of 6 patients, resulting in a total of 4 MLNs instead of 1. The difference between having a N0 (no affected LNs regionally) or N1 status (affected LNs) when confirmed pathologically, can affect postoperative therapy (e.g. adjuvant radiotherapy) and clinical follow-up schedule for the patient. At least 1 out of 6 patients in this study were admitted to a stricter schedule then would have been the case if the pathological report would have noted an pN0 status. One important limitation of the study is that LNs could not be marked intraoperatively, introducing a degree of uncertainty. Nonetheless, the surgeries were conducted with consistent methodology, aiming for *en bloc* resection of LNs clustered together, while particular attention was directed, including through fluorescent imaging, towards areas where avid nodes were identified based on PSMA-PET findings. One patient (subject 5) underwent salvage pelvic lymph node dissection due to a PSMA-PET positive lymph node in the iliac region (see Table 1). Prior to surgical approach to the suspected region, the operating urologists halted the procedure as the observed extensive fibrosis induced by prior therapy posed a substantial risk of iatrogenic damage. Because of the early ending of the procedure, the anatomical area where the lymph node was situated could not be reached and no fluorescent node was identified. This margin of uncertainty was not applicable for other imaging stages besides the surgery, as fluorescent LNs were resected from the LNCs at the pathology department and enclosed in cassettes separate from other nodes. We anticipate that fluorescence imaging with OTL78 may offer significant utility in larger cohorts of patients with PSMA-PET avid LNs who undergo PLND. *Ex vivo* fluorescence imaging, preferably of lymph nodes separated from their clusters, should be considered as the follow-up procedure after intraoperative fluorescence imaging because *in vivo* fluorescent signal can be impaired by overlying adipose or fibrotic tissue, which is almost inevitable with *en bloc* LNC resections. The advantage of *ex vivo* fluorescence imaging with OTL78 in this study was the identification of very small LNs visible on PSMA-PET but missed in primary pathological examination, supporting the patient and the urologist in the choice of follow-up treatment. It is also suggested to perform more frequent sectioning of even the small lymph nodes exhibiting a distinct fluorescence pattern to obtain additional microscopic slides, thereby minimizing the risk of undetected metastases. Although the *ex vivo* results of the individual nodes in this study were promising, the *ex vivo* LNC fluorescence imaging results were excluded from this manuscript, as the high background signal made accurate evaluation challenging. A longer interval between administration and imaging may potentially resolve this issue[18]. For future studies, a 24-h interval is proposed using the lower (0.03 mg/kg) OTL78 dose, given the strong retention of OTL78 in PSMA-overexpressing prostate cancer cells and the expected lymphatic clearance of unbound agent. The potential influence of androgen deprivation therapy (ADT) on PSMA expression was not addressed in this study. Nonetheless, this may be an important consideration for future research as well, as ADT has been suggested to upregulate PSMA expression and could therefore enhance tumor detectability with PSMA-targeted imaging modalities[21].

## Conclusions

In patients with prostate cancer undergoing PLND for staging or salvage purposes, the use of fluorescence imaging identified *ex vivo* all malignant areas showing the feasibility of this approach. Future studies involving fluorescence imaging with OTL78 should be done with larger sample sizes to establish the efficacy of *in vivo* imaging of MLNs.

## Data Availability

As per ethics committee agreements, data collected for the study are not available. This includes participant data and a data dictionary defining each field in the dataset. Deidentified individual participant data can only be shared upon request to researchers with appropriate proposals under the terms of a signed Data Access Agreement. Requests should be directed to the corresponding author.
